# Fasting Serum Bile Acid Profiles in Dogs with Food-Responsive and Immunosuppressant-Responsive Chronic Inflammatory Enteropathy: An Exploratory LC–MS/MS Study

**DOI:** 10.3390/ani16182887

**Published:** 2026-09-14

**Authors:** Gergely Kiss, Borbála Mózes, Ágnes Sterczer, Katalin Lányi, Krisztián Németh, Roland Psáder

**Affiliations:** 1 Department of Internal Medicine, University of Veterinary Medicine Budapest, István u. 2, H-1078 Budapest, Hungary; kiss.gergely@univet.hu (G.K.); sterczer.agnes@univet.hu (Á.S.); psader.roland@univet.hu (R.P.); 2 Food Chain Laboratory Center, Institute of Food Chain Science, University of Veterinary Medicine Budapest, Marek J. u. 2, H-1078 Budapest, Hungary; lanyi.katalin@univet.hu; 3 Department of Physiology and Biochemistry, University of Veterinary Medicine Budapest, István u. 2, H-1078 Budapest, Hungary; nemeth.krisztian@univet.hu

**Keywords:** chronic inflammatory enteropathy, bile acids, food-responsive enteropathy, immunosuppressant-responsive enteropathy, liquid chromatography–tandem mass spectrometry, primary-to-secondary bile acid ratio, dogs

## Abstract

Bile acid (BA) dysmetabolism is implicated in canine chronic inflammatory enteropathy (CIE), but fasting serum profiles across treatment-response groups remain poorly characterized. This exploratory observational study analyzed pretreatment serum from dogs subsequently classified as food-responsive enteropathy (FRE; *n* = 8) or immunosuppressant-responsive enteropathy (IRE; *n* = 5), with four unmatched purpose-bred Beagle controls. Targeted LC–MS/MS, performed using a published canine serum method, provided results for 15 BA species. All samples were analyzed in one batch by an analyst blinded to subsequent group assignment. Eight species quantifiable in every dog and ten derived variables were compared using Kruskal–Wallis tests with Benjamini–Hochberg correction across 18 omnibus tests. Cholic acid and unconjugated primary BA concentrations were higher in IRE than in controls and FRE. The total primary-to-secondary BA ratio showed a monotonic group ordering (control < FRE < IRE; H = 13.76, *p* = 0.0010, q = 0.0092; epsilon-squared = 0.840), with all Bonferroni-corrected pairwise comparisons significant. A post hoc four-scenario sensitivity analysis comprising the submitted zero-substitution analysis and three LOD/LOQ-based alternatives reproduced the same 18-test FDR-significant set and the same ratio inference. This analysis tests robustness to censored-value handling but is not independent validation. The findings suggest group-associated serum BA patterns, but the small sample, clinical heterogeneity, and unmatched controls preclude diagnostic or treatment-predictive interpretation.

## Simple Summary

Chronic inflammatory enteropathy in dogs may improve with dietary treatment alone, whereas some dogs require immunosuppressant medication. Bile acids aid digestion and also participate in intestinal microbial and immune interactions. We measured 12–24 h fasting serum concentrations of individual bile acids in four healthy dogs, eight dogs that later responded to diet, and five dogs that later required prednisolone. The relative circulating concentrations of primary and secondary bile acids differed among groups, and cholic acid concentrations were highest in the group requiring prednisolone. The study was small, and the healthy dogs were younger purpose-bred Beagles maintained under different conditions from the clinical dogs. These findings do not provide a diagnostic test or prove that bile acid measurements predict treatment response. They suggest a group-associated serum pattern that should be examined in larger studies using matched controls and repeated sampling before and after treatment.

## 1. Introduction

Chronic inflammatory enteropathy (CIE) in dogs encompasses a heterogeneous group of disorders characterized by persistent or recurrent gastrointestinal signs after exclusion of diseases that independently explain the presentation [1]. CIE is commonly classified according to treatment response, including food-responsive enteropathy (FRE), immunosuppressant-responsive enteropathy (IRE), and non-responsive enteropathy, although these categories do not necessarily represent discrete mechanisms [1,2]. Bile acid (BA) dysmetabolism has emerged as a potential link among intestinal inflammation, microbial dysbiosis, epithelial dysfunction, and altered immune signaling in canine CIE [3,4,5,6,7].

Bile acids have both digestive and signaling functions. In addition to facilitating lipid absorption, they activate nuclear receptors, particularly farnesoid X receptor (FXR), and membrane receptors such as TGR5 (GPBAR1) [4,8,9,10]. Free secondary BAs, especially deoxycholic acid (DCA), show stronger bactericidal activity than several primary BAs in experimental systems, although most direct evidence is not canine in vivo evidence [11,12,13]. In dogs, cholic acid (CA) and chenodeoxycholic acid (CDCA) are synthesized hepatically and conjugated predominantly with taurine before secretion into the intestine [14,15,16]. Most secreted BAs are actively reabsorbed in the terminal ileum, whereas a smaller fraction reaches the colon and undergoes microbial transformation, including conversion of CA to DCA and CDCA to lithocholic acid (LCA) by 7α-dehydroxylating bacteria such as *Peptacetobacter hiranonis* [17,18,19,20]. Taurine-conjugated species predominate in canine serum, and taurocholic acid (TCA) is commonly the most abundant individual circulating BA [16,21].

Dogs with CIE often show altered fecal BA profiles, including relative enrichment of primary BAs and depletion of secondary BAs, particularly in association with dysbiosis [3,5,11,22]. Expanded fecal profiling has also identified alterations in iso- and oxo-BA species [23]. Proposed contributors include impaired ileal transport, reduced abundance or activity of BA-transforming bacteria, and altered FXR-FGF19 feedback affecting host BA synthesis and transport [6,8,18,19,24].

FXR and TGR5 provide plausible links between BA homeostasis and intestinal inflammation. FXR regulates BA synthesis, transport, epithelial barrier function, and metabolic pathways, whereas TGR5 activation by secondary BAs can modulate macrophage and enteroendocrine signaling, gastrointestinal motility, and inflammatory responses [9,18,24,25,26,27,28]. Most mechanistic evidence linking FXR/TGR5 signaling to intestinal inflammation derives from non-canine or in vitro systems; direct canine in vivo causal evidence remains limited, and serum BA concentrations cannot be interpreted as direct measures of receptor activity or microbial function.

Relative fecal enrichment of primary bile acids and depletion of secondary bile acids have been used to characterize impaired microbial bile acid transformation in canine enteropathy [3,5,11,22,29]. Longitudinal studies indicate that clinical improvement after diet, corticosteroid treatment, or fecal microbiota transplantation may be accompanied by recovery of fecal secondary BAs and microbial function [5,11,30]. Bile acid sequestrants have also been used in selected dogs with chronic enteropathy, although evidence remains limited [31]. By contrast, the biological meaning of a serum primary-to-secondary ratio is less direct because circulating concentrations integrate hepatic synthesis, conjugation, intestinal deconjugation, absorption, enterohepatic cycling, and clearance. Targeted LC–MS/MS can quantify individual canine serum BA species and derived pools [16,21,32,33].

The present study was motivated by a preceding duodenal mucosal RNA-seq analysis performed in the same eight FRE and four control dogs [7]. That study identified enrichment of the KEGG bile secretion pathway and coordinated changes in BA-axis transcripts. The five IRE dogs were not part of that RNA-seq study. The current analysis therefore addresses a distinct biological matrix and analytical focus: fasting circulating BA profiles, with exploratory extension to IRE. It was not designed as a formal transcript–metabolite validation study. We hypothesized that fasting serum BA profiles, including indices of primary-to-secondary BA balance, would differ among controls, FRE dogs, and IRE dogs.

## 2. Materials and Methods

### 2.1. Study Design, Clinical Characterization, and Sample Collection

This was an exploratory observational analysis of pretreatment serum from a clinically assembled cohort; the cohort was not designed or powered specifically for serum BA analysis. Client-owned dogs with gastrointestinal signs persisting for at least 3 weeks and referred for upper or combined upper–lower gastrointestinal endoscopy at the University of Veterinary Medicine Budapest were prospectively enrolled. The diagnostic work-up included clinical examination, Canine Inflammatory Bowel Disease Activity Index (CIBDAI) scoring, hematology, serum biochemistry including electrolytes and routine hepatobiliary variables, canine trypsin-like immunoreactivity (cTLI), canine pancreatic lipase immunoreactivity (cPLI), cortisol measurement, urinalysis, abdominal ultrasonography, fecal parasitology, and *Giardia* antigen testing. Urine protein-to-creatinine ratios were measured in the two hypoalbuminemic IRE dogs to assess renal protein loss. Based on these investigations, no extraintestinal, infectious, parasitic, pancreatic, endocrine, hepatobiliary, renal, metabolic, or neoplastic disorder was identified that independently explained the gastrointestinal signs. Before sampling, the clinical dogs consumed non-therapeutic commercial diets and had not undergone a previous structured therapeutic dietary trial; specifically, no hypoallergenic dietary trial had been instituted. Product-level details, nutrient composition including fat content, treats, supplements, and complementary feeds were not consistently available. The hypoallergenic diets used for response classification were instituted only after baseline sampling. No dog had received pharmacological treatment for the presenting gastrointestinal disease, including antibiotics, glucocorticoids, other immunosuppressive agents, ursodeoxycholic acid, or bile acid sequestrants. Gastrointestinal biopsy specimens from the clinical dogs were evaluated histopathologically according to World Small Animal Veterinary Association criteria as part of the diagnostic work-up.

After a recorded 12–24 h fasting interval, blood was obtained by cephalic venous cannulation into serum separator tubes, allowed to clot for 1 h at room temperature, and centrifuged for 10 min. Exact dog-level fasting durations within this range were not retained. Separated serum was stored at −80 °C until analysis. All serum samples underwent a single freeze–thaw cycle before LC–MS/MS analysis. Samples were collected before any therapeutic dietary or pharmacological intervention, and treatment-response classification was assigned during 6–24 weeks of follow-up. Dogs were classified as FRE when clinical remission was achieved with a hypoallergenic diet (a novel-protein or hydrolyzed-protein diet fed exclusively) maintained for at least 1 month. Dogs were classified as IRE when at least three hypoallergenic dietary trials, all instituted after baseline sampling, had failed to induce clinical remission and remission was subsequently achieved during prednisolone treatment at 1–2 mg/kg/day for at least 1 month while a hypoallergenic diet was continued. In both groups, clinical remission was defined as resolution or sustained marked improvement in the presenting gastrointestinal signs. Thus, BA measurements represented the untreated baseline state, whereas dietary trials, pharmacological treatment, and treatment-response classification occurred subsequently. Four clinically healthy purpose-bred Beagles without a history or clinical evidence of gastrointestinal disease served as controls and were sampled and processed using the same protocol. Individual demographics, leading clinical signs, disease duration, baseline CIBDAI score, and serum albumin, globulin, and cholesterol concentrations are provided in Supplementary Table S1. No ultrasonographic hepatobiliary abnormality or clinically relevant parasitic infection was identified in the clinical cohort.

### 2.2. LC–MS/MS Analysis

Targeted serum BA profiling was performed according to the previously published canine serum LC–MS/MS method of Lányi et al. [32]. Briefly, 500 µL of serum was spiked with an isotope-labeled internal-standard mixture and processed by protein precipitation followed by solid-phase extraction. A BA-depleted serum pool prepared from healthy dogs independent of the 17 study dogs served as the matrix for calibrators and spiked quality-control samples; calibration standards and low- and high-concentration QC spikes were added after depletion. Low (2 µM) and high (100 µM) QC samples were evaluated with an acceptance criterion of ±15%. Chromatographic separation was performed using a Shimadzu Nexera ultra-high-performance liquid chromatography (UHPLC) system coupled to a Shimadzu LCMS-8030+ triple-quadrupole mass spectrometer (Shimadzu Corporation, Kyoto, Japan), with a Kinetex C18 column (Phenomenex, Torrance, CA, USA) and a 14 min gradient, electrospray ionization in both polarities, and multiple-reaction monitoring. Quantification used internal-standard calibration with 1/C^2^ weighted regression. All study samples were analyzed within a single analytical batch, and the analyst was blinded to subsequent clinical group assignment. The finalized analytical output contained results for 15 BA species.

Each sample–analyte combination was injected three times. Sample-level means were used in subsequent analyses, whereas standard deviations and relative standard deviations described injection repeatability of the same final extract. These technical replicates do not represent independent serum preparations or extractions. The laboratory output classified results as quantifiable, non-quantifiable (n.q.), or non-detected (n.d.). Individual injection results, sample-level means, injection-repeatability summaries, analytical status codes, and derived variables are provided in Supplementary Table S2.

### 2.3. Statistical Analysis

Statistical analyses were performed in Python 3.12.13 using SciPy 1.17.0. Because the groups were small and unequal (controls *n* = 4, FRE *n* = 8, IRE *n* = 5), non-parametric methods were used. Continuous variables are summarized as medians and interquartile ranges in the main text; means and standard deviations are provided in the Supplementary Files. No sample-size calculation was performed, and all analyses were exploratory.

Derived variables were calculated per dog using the following operational definitions. Total primary BAs comprised CA, TCA, glycocholic acid, CDCA, taurochenodeoxycholic acid, and glycochenodeoxycholic acid. Total secondary BAs comprised DCA, taurodeoxycholic acid, glycodeoxycholic acid, LCA, taurolithocholic acid, glycolithocholic acid, ursodeoxycholic acid (UDCA), tauroursodeoxycholic acid (TUDCA), and glycoursodeoxycholic acid; UDCA and its conjugates were grouped operationally with secondary-derived BAs for pool calculations. Unconjugated primary BA concentration was CA + CDCA, and taurine-conjugated primary BA concentration was TCA + taurochenodeoxycholic acid. Unconjugated secondary BA concentration was DCA + LCA + UDCA, and taurine-conjugated secondary BA concentration was taurodeoxycholic acid + taurolithocholic acid + TUDCA. The total primary-to-secondary ratio was total primary BAs divided by total secondary BAs. The taurine-conjugated primary-to-secondary ratio was (TCA + taurochenodeoxycholic acid)/(taurodeoxycholic acid + taurolithocholic acid + TUDCA). The taurine conjugation ratio was the proportion of all measured BAs present in taurine-conjugated form.

The laboratory-provided analytical status was retained for each sample–analyte result. For calculation of derived pools, results classified as non-quantifiable (n.q.) or non-detected (n.d.) were assigned a computational value of zero in the submitted analysis; this convention does not imply true biological absence. In response to peer review, a post hoc censoring sensitivity analysis evaluated four data-handling scenarios for all ten derived variables: (i) the submitted zero-substitution analysis, n.d. = 0 and n.q. = 0; (ii) lower bound, n.d. = 0 and n.q. = LOD; (iii) half-limit imputation, n.d. = LOD/2 and n.q. = LOQ/2; and (iv) upper bound, n.d. = LOD and n.q. = LOQ. Matrix-specific limits were obtained from the published method validation [32]. These are operational threshold scenarios, not reconstructed measured concentrations. Within each scenario, the original eight fully quantifiable individual-analyte tests were retained, the ten derived-variable tests were recalculated, and Benjamini–Hochberg adjustment was reapplied across the same family of 18 omnibus tests. This framework tests whether the numerical handling of censored observations changes the inferential pattern without changing the analyte definitions. The taurine-conjugated primary-to-secondary ratio, (TCA + TCDCA)/(TDCA + TLCA + TUDCA), remained one of the original ten derived variables. Although all five of its components were quantifiable in every dog, it was not treated as a sensitivity analysis because it represents a conjugation-specific endpoint rather than an alternative treatment of censored values in the total pools.

Overall three-group comparisons were performed with the Kruskal–Wallis H test for the eight BA species quantifiable in every dog and for ten derived variables. The resulting 18 omnibus *p* values were adjusted using the Benjamini–Hochberg false-discovery-rate procedure, with q < 0.05 considered significant. For variables meeting this threshold, two-sided exact Mann–Whitney U tests were used for the three pairwise comparisons, with Bonferroni correction within each variable. Kruskal–Wallis epsilon-squared was reported as the omnibus effect-size estimate for FDR-significant variables and interpreted descriptively. Exploratory two-sided Spearman correlations assessed age and body weight in relation to the total primary-to-secondary ratio in the full cohort and in the combined clinical population. No multivariable adjustment, receiver-operating-characteristic analysis, cut-off estimation, predictive modeling, or post hoc power analysis was attempted because of the sample size and structural differences between the purpose-bred control group and client-owned clinical groups.

## 3. Results

### 3.1. Cohort Characteristics and Clinical Context

All 17 dogs had pretreatment serum collected after a recorded 12–24 h fasting interval. Baseline CIBDAI scores had a median [interquartile range] of 6 [5–7.25] in FRE and 9 [8,9] in IRE. Serum albumin concentrations ranged from 2.4 to 3.2 g/dL in FRE and from 1.1 to 3.4 g/dL in IRE. None of the FRE dogs was hypoalbuminemic; two IRE dogs were hypoalbuminemic and were considered to have presumptive inflammatory protein-losing enteropathy after renal and other extraintestinal causes of protein loss had been excluded. One had albumin 1.8 g/dL, cholesterol 108 mg/dL, CIBDAI 10, 2 months of clinical signs, and a total primary-to-secondary ratio of 8.858; the other had albumin 1.1 g/dL, cholesterol 250 mg/dL, CIBDAI 9, 1 month of clinical signs, ascites, and a ratio of 6.490. Both achieved clinical remission during prednisolone treatment while a hypoallergenic diet was continued. The three normoalbuminemic IRE dogs had albumin 2.8–3.4 g/dL, cholesterol 187–221 mg/dL, CIBDAI 5–9, disease duration from 6 months to 1 year, and ratios of 6.292–8.273. No dog had a biochemical or ultrasonographic pattern indicating clinically relevant primary hepatobiliary disease. Individual clinical data are reported in Supplementary Table S1. These descriptive data were not used for subgroup inference or adjusted modeling.

Histopathology was performed as part of the clinical diagnostic work-up rather than as a predefined study endpoint. Findings were heterogeneous, predominantly mild, and non-specific, including variable villus shortening, occasional mild lamina propria lymphocyte/plasma-cell infiltration, and sporadic crypt or lacteal changes. No consistent histopathologic pattern defined either treatment-response group; therefore, histologic findings were not subjected to group-level inference or association analyses with serum BA variables [1,34].

### 3.2. Analytical Results, Quantification Status, and Injection Repeatability

Serum BA profiling was completed in all 17 dogs. The analytical dataset contained results for 15 primary and secondary, conjugated and unconjugated BA species. Eight species were quantifiable in every dog and were included in individual-analyte comparisons: CA, TCA, CDCA, taurochenodeoxycholic acid, DCA, taurodeoxycholic acid, taurolithocholic acid, and TUDCA. LCA was quantifiable in 10/17 dogs and UDCA in 5/17 dogs; these analytes were not tested individually but were retained in derived pools using the stated computational zero-substitution approach. None of the five glycine-conjugated species was quantifiable. Across 255 sample–analyte observations, 151 were quantifiable, 69 were non-quantifiable, and 35 were non-detected. Among species with quantitative results, median analyte-specific injection RSDs ranged from 2.87% to 11.15%. The maximum injection RSD was 18.45% for one taurolithocholic acid result; all other quantifiable sample–analyte means had RSDs of 14.21% or less. Analytical results and injection-repeatability data for the present cohort are provided in Supplementary Table S2.

The absence of quantifiable glycine-conjugated BAs was biologically compatible with the strong predominance of taurine conjugation reported in dogs, but non-quantification should not be interpreted as confirmed biological absence [15,16,35,36]. In the validated canine serum method, matrix-specific limits of detection/quantification (LOD/LOQ) were 8.3/25 nM for glycocholic acid, 8.3/25 nM for glycochenodeoxycholic acid, 1.7/5 nM for glycodeoxycholic acid, 4.2/12.5 nM for glycolithocholic acid, and 4.2/12.5 nM for glycoursodeoxycholic acid [32].

### 3.3. Individual Bile Acid Concentrations

**Figure 1:**
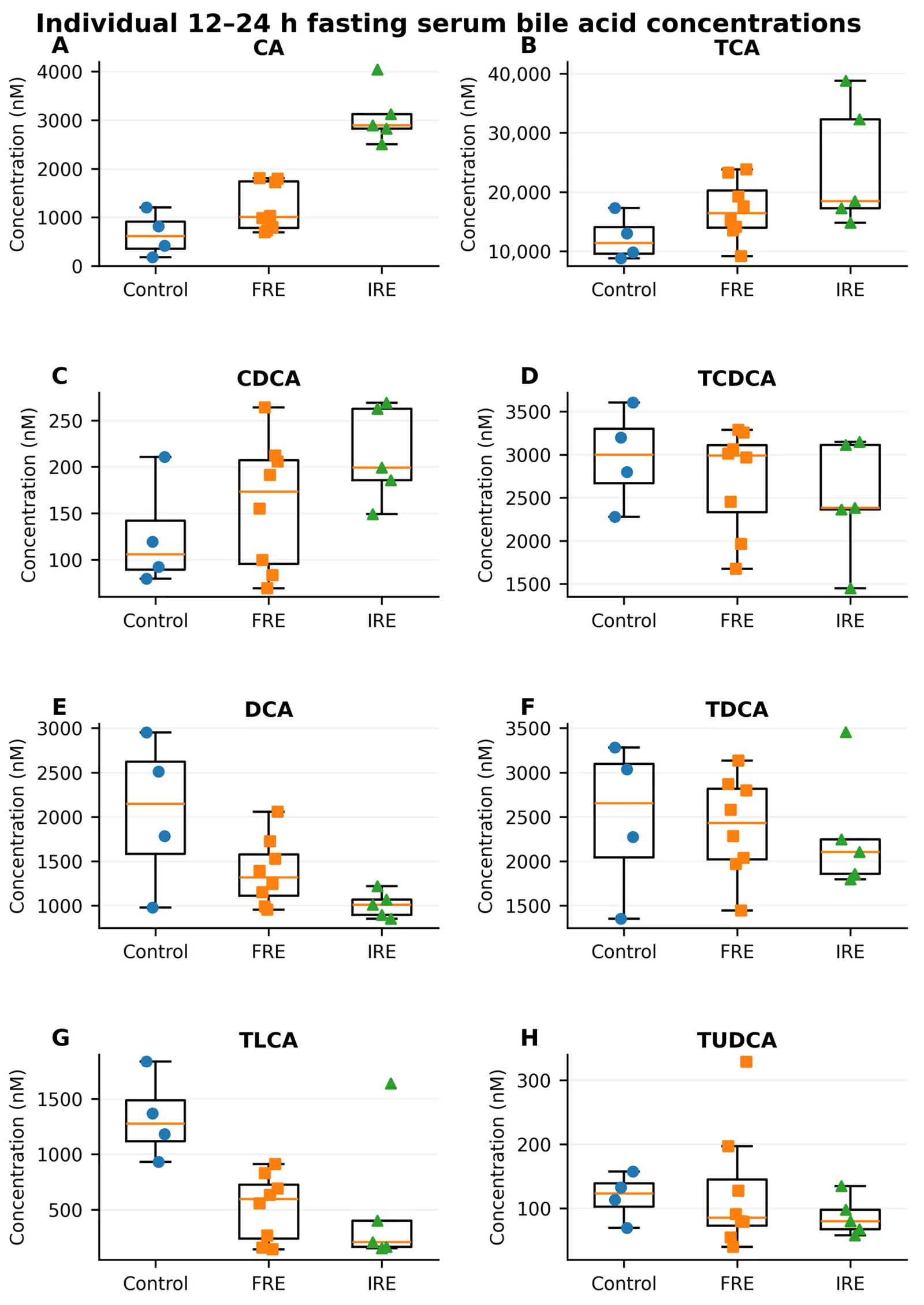
Individual 12–24 h fasting serum concentrations of eight bile acid species quantifiable in every dog: healthy controls (*n* = 4; blue circles), dogs with food-responsive enteropathy (FRE; *n* = 8; orange squares), and dogs with immunosuppressant-responsive enteropathy (IRE; *n* = 5; green triangles). Panels show (**A**) cholic acid (CA), (**B**) taurocholic acid (TCA), (**C**) chenodeoxycholic acid (CDCA), (**D**) taurochenodeoxycholic acid (TCDCA), (**E**) deoxycholic acid (DCA), (**F**) taurodeoxycholic acid (TDCA), (**G**) taurolithocholic acid (TLCA), and (**H**) tauroursodeoxycholic acid (TUDCA). Boxes indicate interquartile ranges, horizontal lines indicate medians, whiskers extend to the most extreme value within 1.5 times the interquartile range, and individual observations are overlaid. Complete statistical results are provided in Supplementary Table S3.

Concentrations of the eight BA species quantifiable in every sample are shown in Figure 1. Complete descriptive and inferential results are provided in Supplementary Tables S2 and S3.

#### 3.3.1. Primary Bile Acids

CA concentrations showed a monotonic group ordering, with medians [interquartile range] of 615 [356–912] nM in controls, 1010 [782–1742] nM in FRE, and 2895 [2827–3122] nM in IRE. The overall difference remained significant after FDR correction (H = 10.94, raw p = 0.0042, q = 0.0189; epsilon-squared = 0.639). IRE dogs had higher CA concentrations than controls (Bonferroni-corrected p = 0.0476) and FRE dogs (*p* = 0.0047), whereas FRE and controls did not differ. TCA was the predominant measured BA species in all groups. No other individual primary BA remained significant after FDR correction.

#### 3.3.2. Secondary Bile Acids

DCA and taurolithocholic acid concentrations were descriptively lower in the CIE groups than in controls, but no individual secondary BA remained significant after FDR correction. For taurolithocholic acid, the raw omnibus *p* value was 0.0327 and the adjusted q value was 0.1177. Taurodeoxycholic acid and TUDCA showed no evidence of group differences.

### 3.4. Derived Bile Acid Variables

**Figure 2:**
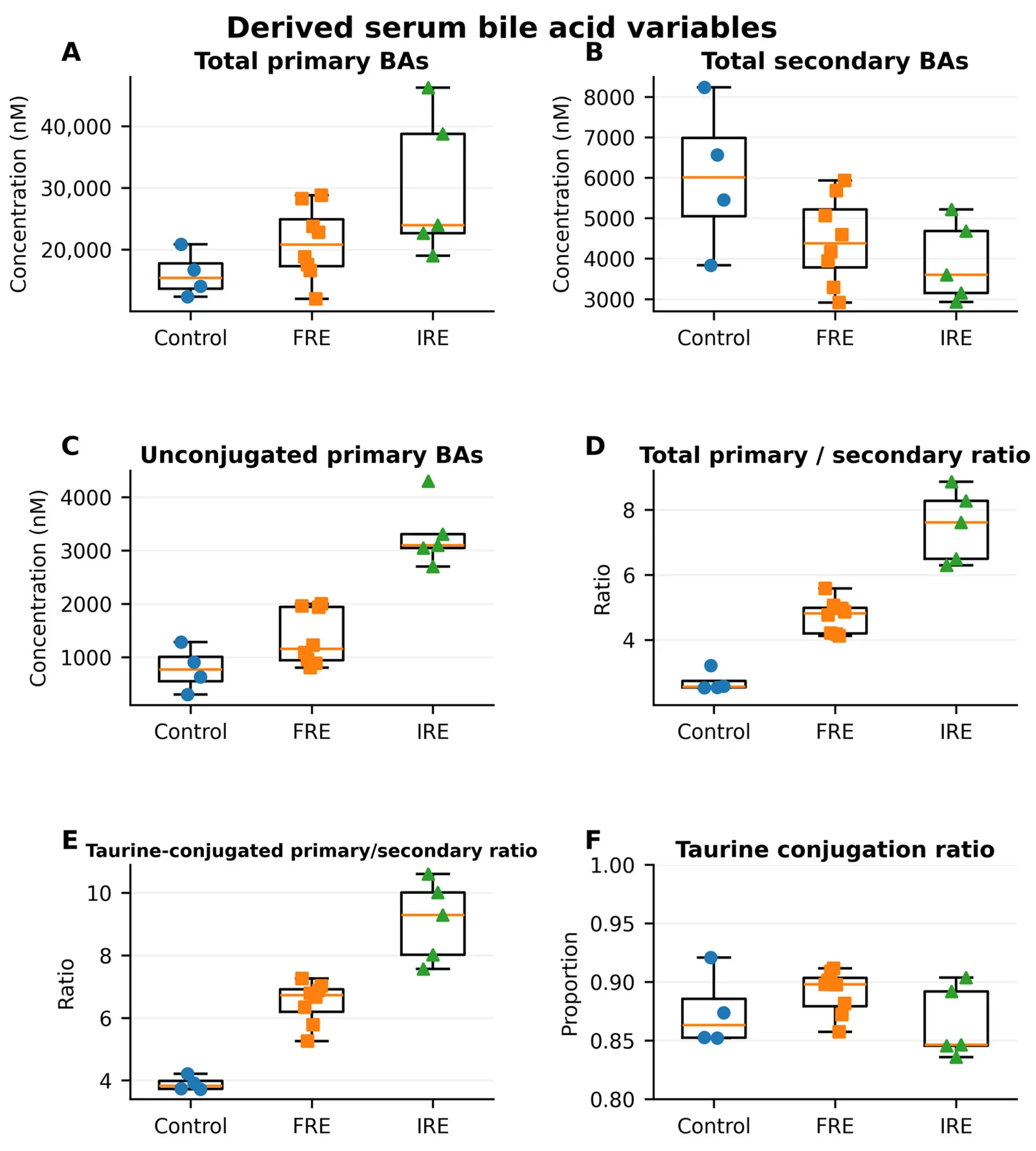
Selected derived 12–24 h fasting serum bile acid variables in healthy controls (*n* = 4; blue circles), dogs with food-responsive enteropathy (FRE; *n* = 8; orange squares), and dogs with immunosuppressant-responsive enteropathy (IRE; *n* = 5; green triangles). Panels show (**A**) total primary bile acid concentration, (**B**) total secondary bile acid concentration, (**C**) unconjugated primary bile acid concentration (CA + CDCA), (**D**) the total primary-to-secondary bile acid ratio, (**E**) the taurine-conjugated primary-to-secondary bile acid ratio, and (**F**) the taurine conjugation ratio, defined as the proportion of all measured bile acids present in taurine-conjugated form. Boxes indicate interquartile ranges, horizontal lines indicate medians, whiskers extend to the most extreme value within 1.5 times the interquartile range, and individual observations are overlaid. Complete statistical results are provided in Supplementary Table S3.

Selected derived serum BA variables are visualized in Figure 2; complete results for all derived variables are provided in Supplementary Tables S2 and S3.

#### 3.4.1. Total Primary, Secondary, and Measured Bile Acids

Total primary, total secondary, and total measured BA concentrations did not differ significantly among groups after FDR correction. The distributions of total primary and total secondary BA concentrations are shown in Figure 2; complete descriptive statistics, including total measured BA concentration, are provided in Supplementary Table S3.

#### 3.4.2. Unconjugated and Taurine-Conjugated Concentrations and Taurine Conjugation Ratio

Unconjugated primary BA concentration (CA + CDCA) differed among groups after FDR correction (H = 11.19, raw *p* = 0.0037, q = 0.0189; epsilon-squared = 0.657). Median [interquartile range] concentrations were 767 [544–1001] nM in controls, 1155 [939–1942] nM in FRE, and 3096 [3044–3308] nM in IRE. IRE dogs had higher concentrations than controls (Bonferroni-corrected p = 0.0476) and FRE dogs (p = 0.0047), whereas FRE and controls did not differ. Taurine-conjugated primary BA concentration, unconjugated and taurine-conjugated secondary BA concentrations, and the taurine conjugation ratio did not show FDR-significant group differences.

#### 3.4.3. Primary-to-Secondary Bile Acid Ratios

The total primary-to-secondary BA ratio showed a monotonic group ordering, with medians [interquartile range] of 2.56 [2.54–2.74] in controls, 4.81 [4.20–4.99] in FRE, and 7.60 [6.49–8.27] in IRE. The overall difference was significant (H = 13.76, raw *p* = 0.0010, q = 0.0092; epsilon-squared = 0.840), and all three Bonferroni-corrected pairwise comparisons were significant (control vs. FRE, *p* = 0.0121; control vs. IRE, *p* = 0.0476; FRE vs. IRE, *p* = 0.0047). The complete post hoc censoring sensitivity analysis evaluated four data-handling scenarios, recalculated all ten derived variables, and repeated Benjamini–Hochberg adjustment across the same 18 tests within each scenario. For the total primary-to-secondary ratio, control/FRE/IRE medians were 2.560/4.810/7.604 in the submitted zero-substitution analysis, 2.557/4.794/7.556 under the lower-bound scenario, 2.556/4.787/7.532 under half-limit imputation, and 2.552/4.766/7.462 under the upper-bound scenario. Because all dog-level ranks were unchanged, H = 13.765, raw *p* = 0.001026, q = 0.009232, observed epsilon-squared = 0.840, and the three Bonferroni-corrected pairwise *p* values (0.012121, 0.047619, and 0.004662) were unchanged in all four scenarios. The complete FDR-significant set also remained unchanged: CA, unconjugated primary BAs, the total primary-to-secondary ratio, and the taurine-conjugated primary-to-secondary ratio. The latter remained an original conjugation-specific derived variable, not a sensitivity analysis. The three normoalbuminemic IRE dogs also had total primary-to-secondary ratios above the complete FRE range; this descriptive observation was not subjected to a separate inferential test. Key FDR-significant results are summarized in Table 1.

### 3.5. Exploratory Associations of Age and Body Weight with the Primary-to-Secondary Ratio

Exploratory Spearman analyses did not identify statistically significant associations of age or body weight with the total primary-to-secondary ratio in either the full cohort or the combined FRE–IRE population; complete coefficients and *p* values are reported in Supplementary Table S3. These analyses were descriptive and do not constitute covariate adjustment or exclude confounding by age, breed, husbandry, baseline diet, or other structural group differences.

## 4. Discussion

### 4.1. Principal Findings

This exploratory study suggested two closely related serum BA patterns. IRE dogs had a CA-dominated increase in unconjugated primary BA concentration, while the total primary-to-secondary ratio retained the same monotonic ordering from controls to FRE to IRE across the submitted zero-substitution analysis and three LOD/LOQ-based alternative scenarios. Both higher primary and lower secondary pools contributed numerically to the ratio, but only the CA-dominated unconjugated primary increase had independent component-level FDR support. The FDR-significant variables are related features of the same dataset and should not be interpreted as independent findings.

### 4.2. Interpretation of the Serum Primary-to-Secondary Bile Acid Ratio

The primary-to-secondary ratio provided the clearest separation among groups. An increased serum ratio may reflect a larger primary BA pool, a smaller secondary BA pool, or both. In the present data, the increase in CA and unconjugated primary BA concentration in IRE indicates an important numerator contribution, whereas total secondary BA concentration was descriptively lower but not FDR-significant. The four-scenario sensitivity framework addressed one specific question: whether alternative numerical treatment of the 69 n.q. and 35 n.d. observations changed the ten derived variables or the complete 18-test inferential pattern. It did not. This supports robustness to the evaluated censoring assumptions within the supplied dataset, but threshold substitution does not recover the unknown concentrations and does not constitute independent validation or establish specificity for treatment-response phenotype. The taurine-conjugated primary-to-secondary ratio has a different, conjugation-specific biological composition and is therefore interpreted as an original derived endpoint rather than as validation of the total-pool handling.

This serum ratio should not be equated with the fecal primary-to-secondary ratio used to characterize microbial BA transformation. Fecal ratios are more directly influenced by intestinal deconjugation and microbial conversion, whereas serum concentrations integrate host synthesis, transport, absorption, enterohepatic recirculation, and clearance. Consequently, the observed ordering is compatible with altered BA homeostasis but does not identify a specific microbial or host mechanism. Likewise, complete rank separation in a small, unmatched cohort should not be interpreted as clinical validation or as evidence that a baseline serum ratio predicts dietary or prednisolone response.

### 4.3. Elevated Cholic Acid and Unconjugated Primary Bile Acids in IRE

IRE dogs had higher CA and unconjugated primary BA concentrations than controls and FRE dogs. Potential explanations include altered hepatic synthesis, intestinal deconjugation, ileal reabsorption, or enterohepatic clearance. Changes in FXR-FGF19 feedback could contribute, but CYP7A1 activity, ileal transport, bacterial bile salt hydrolase activity, and hepatic clearance were not measured. The result is therefore best regarded as a serum phenotype associated with the treatment-response groups in this cohort, not as evidence for a single causal pathway.

### 4.4. Taurolithocholic Acid

Taurolithocholic acid was descriptively lower in FRE and IRE than in controls, but the difference did not remain significant after correction across the 18 omnibus tests. This observation is therefore hypothesis-generating and should not be interpreted as evidence of altered microbial LCA production.

### 4.5. Taurine Conjugation Ratio

The taurine conjugation ratio did not differ significantly among groups. Because circulating conjugation patterns are influenced by BA synthesis, intestinal processing, enterohepatic cycling, and clearance, this result should not be interpreted as a direct measure of hepatic conjugation capacity.

### 4.6. Limitations

The study was limited by its small, clinically assembled cohort, absence of a sample-size calculation, and the structural differences between young purpose-bred Beagle controls and heterogeneous client-owned clinical dogs. Age, breed, husbandry, baseline diet, disease severity, and other unmeasured factors may therefore have contributed to the observed group patterns. Before biopsy and serum collection, the clinical dogs consumed non-therapeutic commercial diets and had not undergone a previous therapeutic dietary trial, but detailed product composition, fat content, treats, supplements, and complementary feeds were unavailable. The hypoallergenic diets used for response classification were introduced only after baseline sampling. Exact dog-level fasting duration within the recorded 12–24 h interval was also unavailable, so comparability within that interval could not be assessed. The exploratory age and body weight correlations do not constitute covariate adjustment. Effect-size estimates from these small, unequal groups are potentially unstable and should be interpreted as observed cohort descriptors rather than precise population estimates. The four-scenario sensitivity analysis evaluates a transparent range of censoring assumptions but does not recover the unknown true concentrations of n.d. or n.q. observations.

Serum was sampled once before treatment, precluding assessment of longitudinal or within-dog treatment effects. Two IRE dogs had presumptive inflammatory protein-losing enteropathy, including one with ascites, and disease severity or protein loss may have contributed to the IRE phenotype. The three normoalbuminemic IRE dogs also had ratios above the FRE range, but this small descriptive subset cannot separate treatment-response phenotype from disease severity and does not support separate subgroup inference. Histopathologic findings were heterogeneous, predominantly mild, and non-specific; no single lesion defined FRE or IRE, consistent with the limited specificity and imperfect clinical correlation of histopathologic scoring in canine chronic enteropathy [1,34]. No parallel fecal BA, microbiome, bacterial BA-transformation, hepatic synthesis, ileal transport, or clearance measurements were available. The three injections characterized repeatability of the same final extract rather than independent extraction precision. Single-batch analysis by a blinded analyst reduced, but did not eliminate, the possibility of technical bias.

### 4.7. Future Directions

Larger prospective studies should include matched controls, serial pretreatment and post-treatment sampling, and parallel serum, fecal BA, and microbiome analyses. Independent validation is required before serum BA ratios can be evaluated for clinical stratification or monitoring.

## 5. Conclusions

In this small exploratory cohort, pretreatment fasting serum BA profiles suggested a group-associated pattern among unmatched healthy controls and dogs subsequently classified as FRE or IRE. The principal findings were a CA-dominated increase in unconjugated primary BAs in IRE and a monotonic control–FRE–IRE ordering of the total primary-to-secondary ratio that persisted across the submitted zero-substitution analysis and three LOD/LOQ-based alternatives. Both sides of the ratio contributed numerically, but component-level inferential support was strongest for the CA-dominated primary increase. The four-scenario analysis addresses sensitivity to censored-value handling but is not independent validation. The results complement the previously reported mucosal BA-axis transcriptional findings in the shared control–FRE cohort but do not establish a direct transcript–metabolite relationship, a causal mechanism, or a treatment-predictive biomarker. These hypothesis-generating findings require validation in larger matched cohorts.

## Figures and Tables

**Table 1:** Key fasting serum bile acid results by treatment-response group. Values are median [interquartile range]. Concentrations are expressed in nM; ratios are dimensionless. q values are Benjamini–Hochberg-adjusted values from the original family of 18 omnibus tests. Epsilon-squared is the observed rank-based omnibus effect-size estimate.

Variable	Control (*n* = 4)	FRE (*n* = 8)	IRE (*n* = 5)	BH q	Observed ε^2^
Cholic acid (nM)	615 [356–912]	1010 [782–1742]	2895 [2827–3122]	0.0189	0.639
Unconjugated primary BAs (nM)	767 [544–1001]	1155 [939–1942]	3096 [3044–3308]	0.0189	0.657
Total primary-to-secondary ratio	2.56 [2.54–2.74]	4.81 [4.20–4.99]	7.60 [6.49–8.27]	0.0092	0.840
Taurine-conjugated primary-to-secondary ratio	3.83 [3.73–3.99]	6.72 [6.20–6.92]	9.30 [8.02–10.01]	0.0092	0.840

## Data Availability

All individual LC–MS/MS results provided for this cohort, technical injection data, analytical status summaries, derived variables, and statistical results supporting this study are included in the article and Supplementary Tables S1–S3. Further inquiries can be directed to the corresponding author.

## Supplementary Materials

The following supporting information can be downloaded at: https://www.mdpi.com/article/10.3390/ani16182887/s1, Table S1. Demographic and baseline clinical characteristics of dogs included in the study; Table S2. Individual serum BA LC–MS/MS results, technical injection data, analytical status and injection-repeatability summaries, original derived variables, matrix LOD/LOQ values, and individual total primary-to-secondary ratios under the four data-handling scenarios; Table S3. Complete original Kruskal–Wallis results with Benjamini–Hochberg-adjusted q values, epsilon-squared effect sizes, Bonferroni-corrected pairwise comparisons, descriptive statistics, exploratory age and body weight correlations, and the complete four-scenario post hoc censoring sensitivity analysis.

## Abbreviations

The following abbreviations are used in this manuscript:

BA	Bile acid
CA	Cholic acid
CDCA	Chenodeoxycholic acid
CIBDAI	Canine Inflammatory Bowel Disease Activity Index
CIE	Chronic inflammatory enteropathy
cPLI	Canine pancreatic lipase immunoreactivity
cTLI	Canine trypsin-like immunoreactivity
DCA	Deoxycholic acid
FDR	False discovery rate
FRE	Food-responsive enteropathy
FXR	Farnesoid X receptor
IRE	Immunosuppressant-responsive enteropathy
IQR	Interquartile range
LC–MS/MS	Liquid chromatography–tandem mass spectrometry
LCA	Lithocholic acid
n.d.	Non-detected
n.q.	Non-quantifiable
RSD	Relative standard deviation
SD	Standard deviation
TCA	Taurocholic acid
TCDCA	Taurochenodeoxycholic acid
TDCA	Taurodeoxycholic acid
TGR5	Takeda G protein-coupled receptor 5
TLCA	Taurolithocholic acid
TUDCA	Tauroursodeoxycholic acid
UDCA	Ursodeoxycholic acid
UHPLC	Ultra-high-performance liquid chromatography
