# Supplementary material for: Fasting Serum Bile Acid Profiles in Dogs with Food-Responsive and Immunosuppressant-Responsive Chronic Inflammatory Enteropathy: An Exploratory LC–MS/MS Study
Source: Animals (Basel). 2026 Sep 14;16(18):2887. doi: 10.3390/ani16182887 (PMC13603148; doi:10.3390/ani16182887)
Supplement: Supplementary file 1 [file animals-16-02887-s001.zip › Supplementary Table S1.pdf]

Supplementary Table S1. Demographic and baseline clinical characteristics of dogs included in the study. The table reports breed, sex, age, and body weight for eight dogs with food-responsive enteropathy (FRE), five dogs with immunosuppressant-responsive enteropathy (IRE), and four healthy control Beagles. For the clinical dogs, leading gastrointestinal signs, disease duration, baseline Canine Inflammatory Bowel Disease Activity Index (CIBDAI) score, and serum albumin, globulin, and cholesterol concentrations are shown. Before biopsy and baseline serum collection, no clinical dog had received a therapeutic diet for the presenting gastrointestinal disease; detailed baseline diet product, composition including fat content, treats, supplements, and complementary feeds were not systematically retained. All hypoallergenic therapeutic dietary trials used for response classification were instituted only after baseline sampling. The young purpose-bred Beagle controls were not matched to the client-owned clinical dogs.

ID	Breed	Sex	Age (years)	Body weight (kg)	Group	Clinical signs and duration	CIBDAI	Albumin (g/dL) Ref: 2.3-4.1	Globulin (g/dL) Ref: 2.5-4.5	Cholesterol (mg/dL) Ref: 120-270
1	Miniature Poodle	Female, spayed	3	7.2	FRE	vomiting, SBD; 5 months	8	3.1	3.9	198
7	Mixed breed	Male, castrated	9	8.4	FRE	vomiting, SBD, LBD, weight loss; 8 months	5	2.6	3.2	178
9	Staffordshire Bull Terrier	Female, spayed	11	19.9	FRE	vomiting, SBD, LBD; 4 months	5	2.8	**4.8**	226
11	Australian Shepherd	Male, intact	2	20.6	FRE	vomiting, SBD, LBD; 6 months	7	3.2	**4.6**	230
13	German Shepherd	Female, spayed	3	32	FRE	SBD, weight loss; 1 year	6	2.4	3.1	150
15	Havanese	Female, spayed	6	4.7	FRE	vomiting, SBD; 1.5 years	8	2.7	3.7	185
17	Mixed breed	Female, intact	2	23.5	FRE	SBD, weight loss, hyporexia; 4 months	4	2.9	3	217
29	Mixed breed	Male, castrated	6	10.9	FRE	vomiting, SBD, weight loss; 4 months	6	3	4	243

ID	Breed	Sex	Age (years)	Body weight (kg)	Group	Clinical signs and duration	CIBDAI	Albumin (g/dL) Ref: 2.3-4.1	Globulin (g/dL) Ref: 2.5-4.5	Cholesterol (mg/dL) Ref: 120-270
18	Giant Schnauzer	Male, intact	2	38	IRE	vomiting, SBD, LBD; 6 months	5	2.8	3.7	214
19	Boston Terrier	Male, intact	7	12.4	IRE	vomiting, SBD, weight loss; 2 months	10	**1.8**	**1.7**	**108**
22	Tibetan Terrier	Female, intact	10	10.8	IRE	SBD, weight loss, ascites; 1 month	9	**1.1**	3.2	250
32	Havanese	Male, castrated	1	5.5	IRE	vomiting, SBD, weight loss; 1 year	8	3.3	3.3	221
40	Miniature Poodle	Female, spayed	4	6.1	IRE	vomiting, SBD, weight loss; 6 months	9	3.4	2.9	187
24	Beagle	Male, intact	1	12.5	Control					
25	Beagle	Male, intact	1	11.8	Control					
26	Beagle	Female, intact	1	11.2	Control					
27	Beagle	Female, intact	1	9.8	Control					

Serum was collected after a recorded 12–24 h fasting interval before treatment; exact dog-level fasting durations were not retained. Treatment-response classification was assigned during 6–24 weeks of follow-up. FRE indicated remission with a hypoallergenic diet (a novel-protein or hydrolyzed-protein diet fed exclusively). IRE indicated failure of at least three hypoallergenic dietary trials, all instituted after baseline sampling, followed by remission with prednisolone while a hypoallergenic diet was continued. The two hypoalbuminemic IRE dogs had presumptive inflammatory protein-losing enteropathy after renal and other extraintestinal causes were excluded; both achieved remission with prednisolone and diet. Histopathologic findings were heterogeneous and non-specific and were not used as group-defining lesions. SBD, small-bowel diarrhea; LBD, large-bowel diarrhea.
